# Supplementary material for: Structural insights into light-driven anion pumping in cyanobacteria
Source: Nat Commun. 2022 Oct 29;13:6460. doi: 10.1038/s41467-022-34019-9 (PMC9617919; doi:10.1038/s41467-022-34019-9)
Supplement: Supplementary file 1 — Supplementary Information [file 41467_2022_34019_MOESM1_ESM.pdf]

Supplementary Information

Structural insights into light-driven anion pumping in cyanobacteria

Astashkin R. et al.

Supplementary Figures

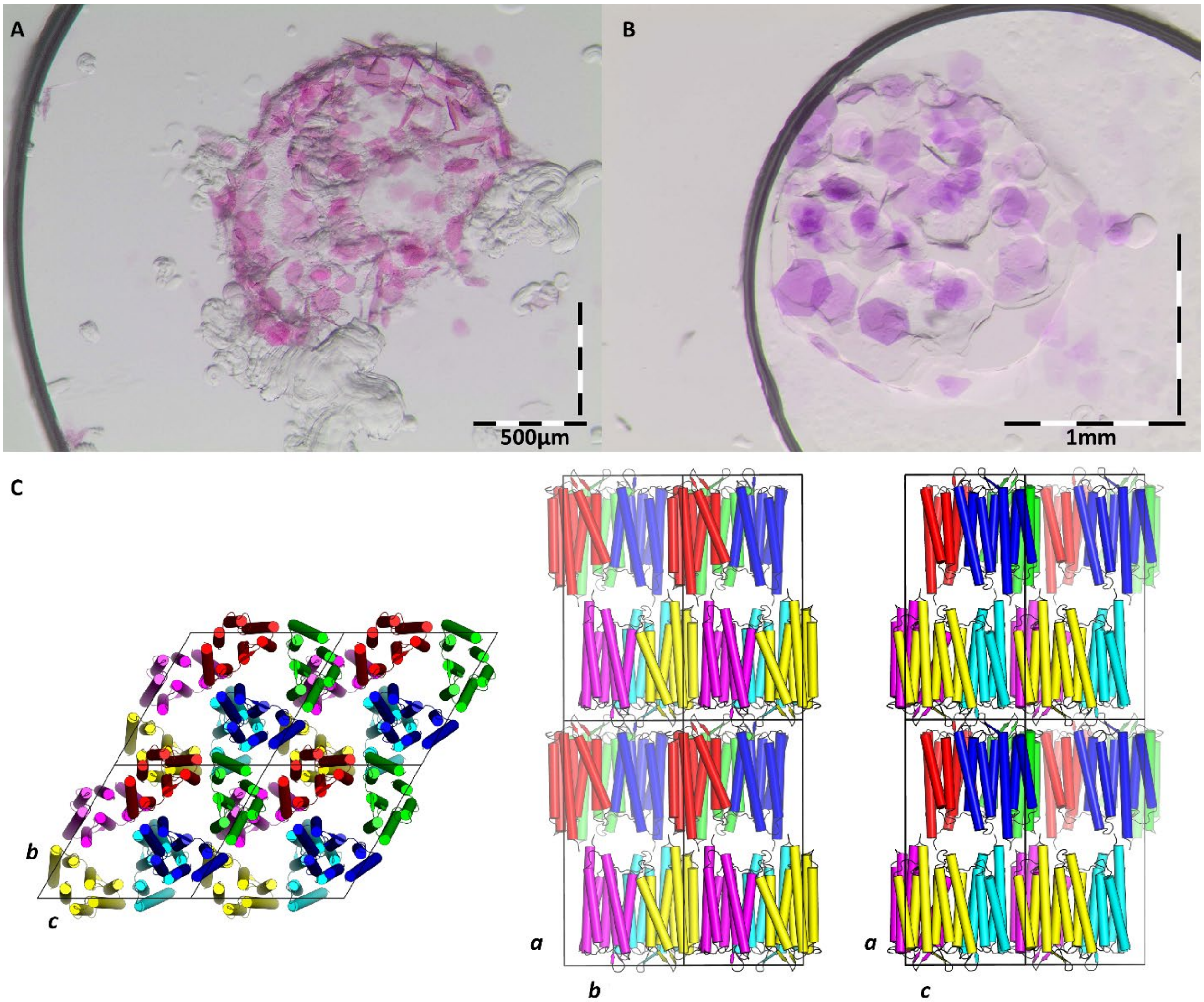

Supplementary Fig. 1 | Crystals of *Sy*HR and crystal packing. (A) Crystals of the red form of *Sy*HR. Best crystals of 6 crystallization trials are shown. (B) Crystals of the violet form of *Sy*HR. Best crystals of 5 crystallization trials are shown. (C) Crystal packing of *Sy*HR.

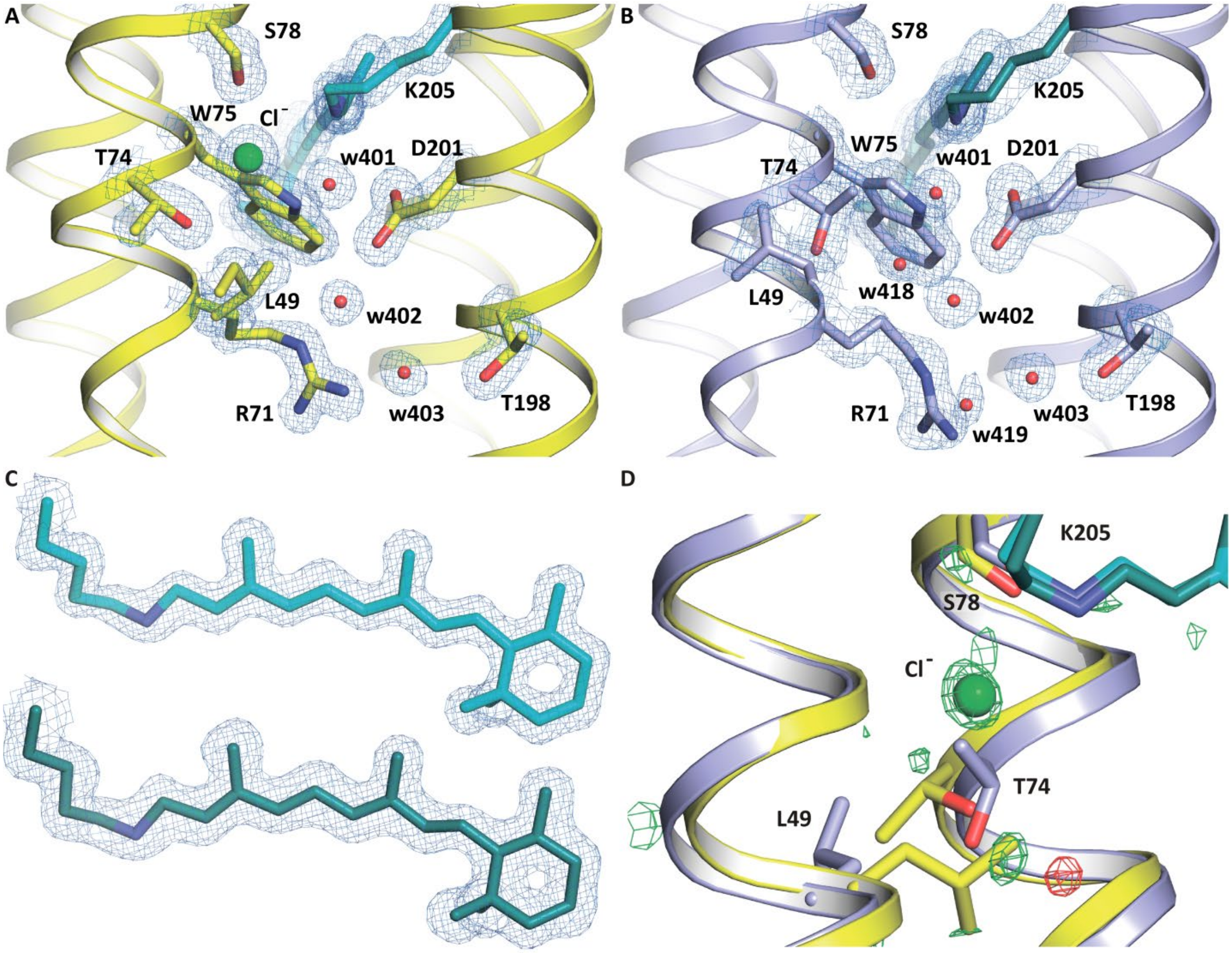

Supplementary Fig. 2 | Comparison of electron density maps for ground and O-states**.** (A) *2Fo-Fc* map of chloride-bound ground state of *Sy*HR, countered at level 1.5sigma (B) *2Fo-Fc* map of O-state of *Sy*HR, countered at level 1.5sigma.. (C) *2Fo-Fc* maps of retinal for ground state (top) and O-state (bottom). (D) Alignment of structures of the O and the ground state (colored blue and yellow respectively). *Fo-Fc* map of the O state structure shows the presence of the low population of the ground state in the data.

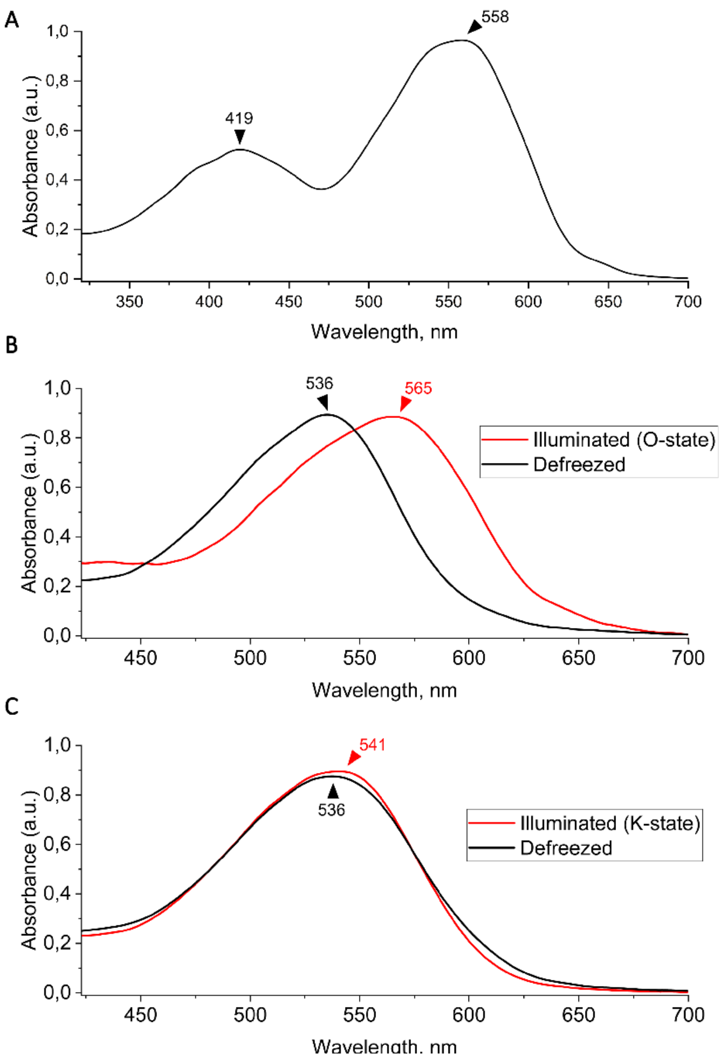

Supplementary Fig. 3 | Absorption spectra of the crystals of *Sy*HR. (A) Absorption of the violet crystals (SO_4^{2-}-form). (B), (C) Relaxation of the spectra of the illuminated crystals after defreezing.

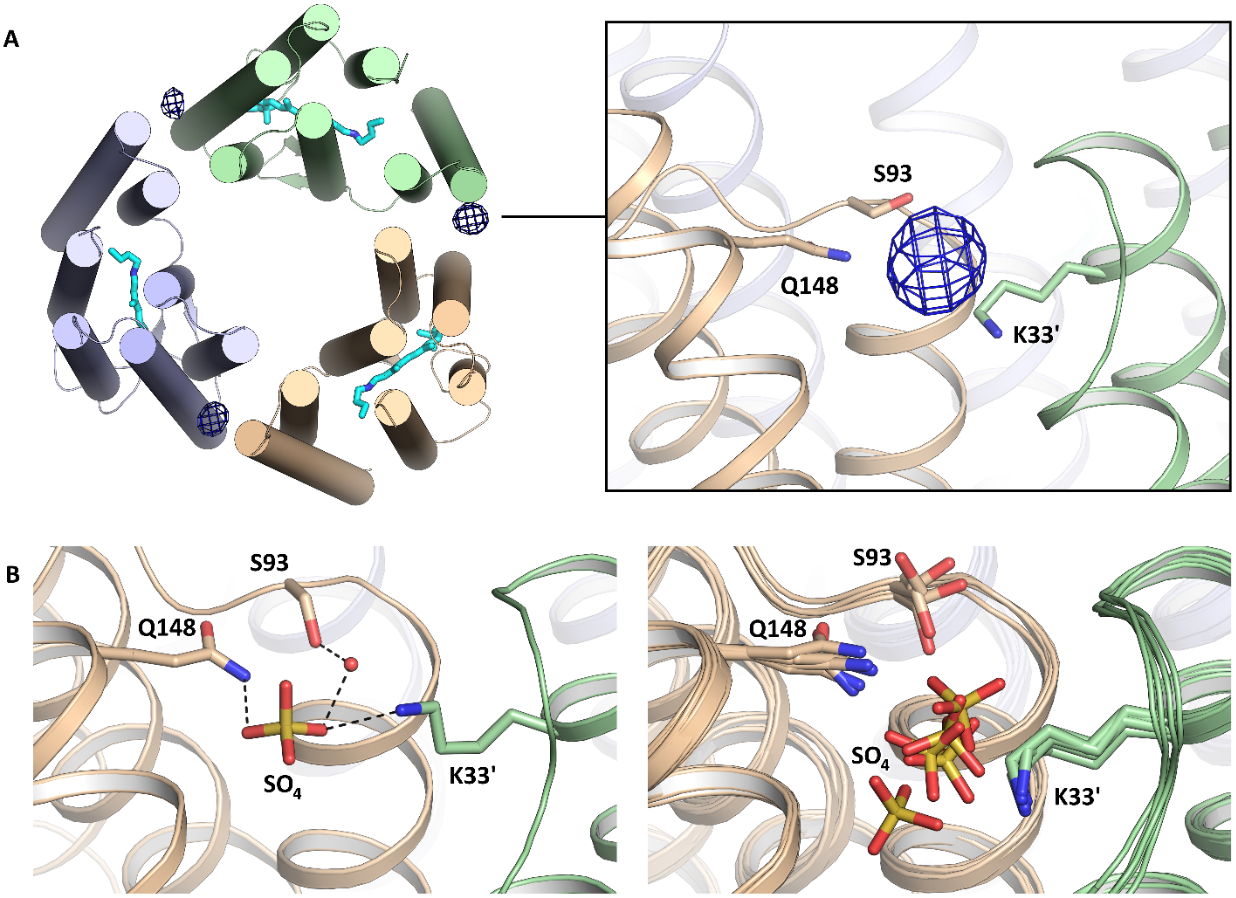

Supplementary Fig. 4 | Molecular dynamics simulation of sulfate-bound *Sy*HR. (A) Molecular modeling of sulfate binding to *Sy*HR trimer. Average density of sulfate ions during 500 ns-long simulation contoured at the level of 0.225 a.m.u./Å^3 is shown as a blue mesh. Enrichment of sulfates is observed at the interface between the protomers, near the site identified in the crystallographic structure. The starting system and the sulfate density data are available as Supplementary Data 1 and 2 respectively. (B) Comparison of the sulfate-binding site in the crystal structure (left) and of the conformations observed in the simulation (right). 5 snapshots taken each 100 ns are shown.

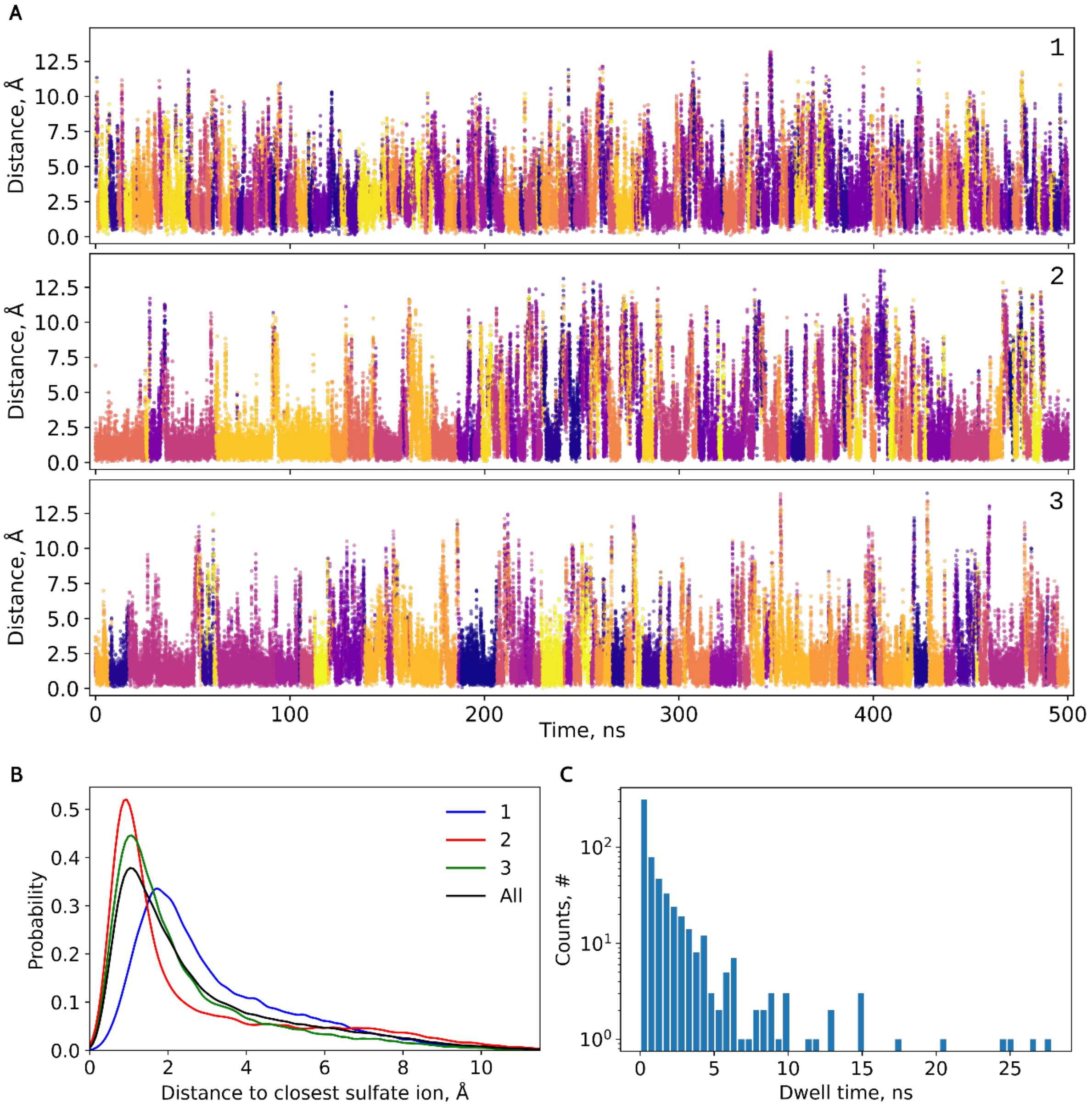

Supplementary Fig. 5 | Ion dynamics in molecular dynamics simulation of sulfate-bound *Sy*HR. (A) Distance from each binding site (Supplementary Fig. 4A) to the sulfur of the closest sulfate ion. Sites 1, 2 and 3 are located at the interfaces between protomers A and B, B and C, and A and C, correspondingly. The plots are colored accordingly to the identifier of the sulfate ion closest to the binding site. (B) Probability distribution of the distances from each binding site to the closest sulfate atom. (C) Distribution of the dwell times for all sites. The ion is considered to be bound if it is closer than 2.5 Å to the binding site, and unbinding is recorded when the ion is further than 2.5 Å from the binding site for at least 500 ps. 304, 130 and 158 binding events are observed for the first, second and third site, correspondingly. The sites are occupied 49, 64 and 68% of the time (average is 60%).

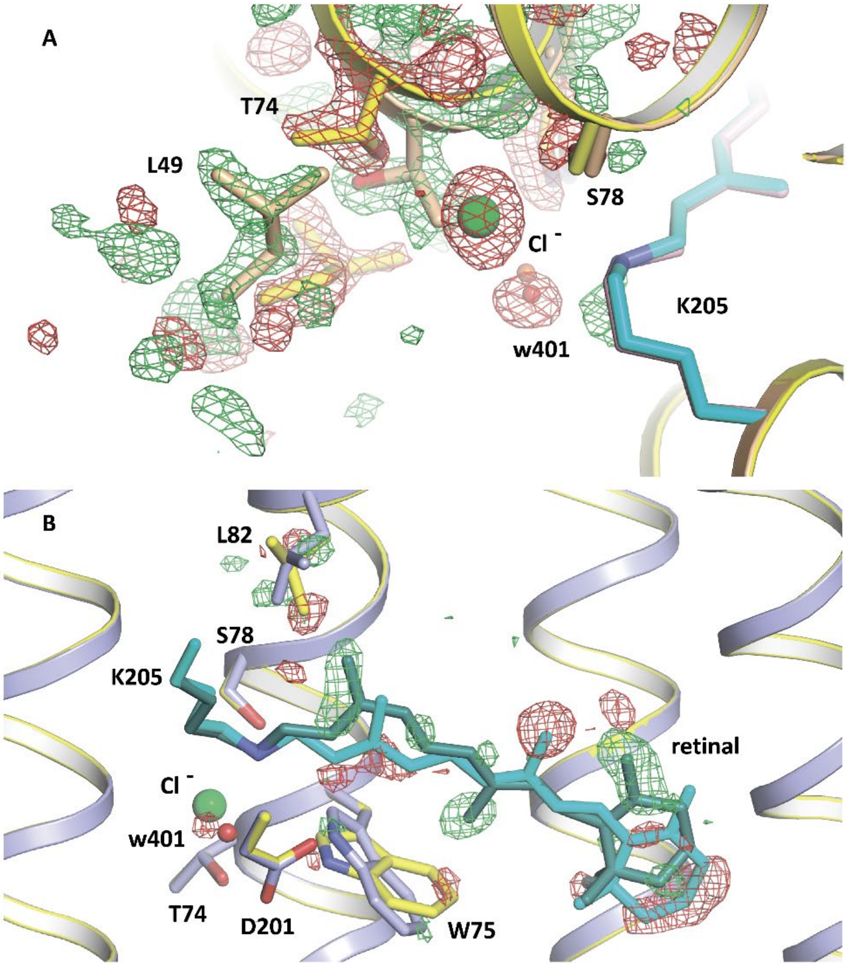

Supplementary Fig. 6 | Examples of the F_{oLight}-F_{oDark} difference electron density of the O and K states of *Sy*HR. (A) F_{oO}-F_{oGr} difference electron densities. Structures of the ground (yellow) and O (orange) states are aligned. The cofactor retinal and K205 are colored teal for the ground state structure of *Sy*HR. (B) F_{oK}-F_{oGr} difference electron densities. Structures of the ground (yellow) and K (light blue) states are aligned. The cofactor retinal and K205 are colored teal and deepteal for the ground and K state structures of *Sy*HR, respectively. The maps are contoured at 4.0σ level.

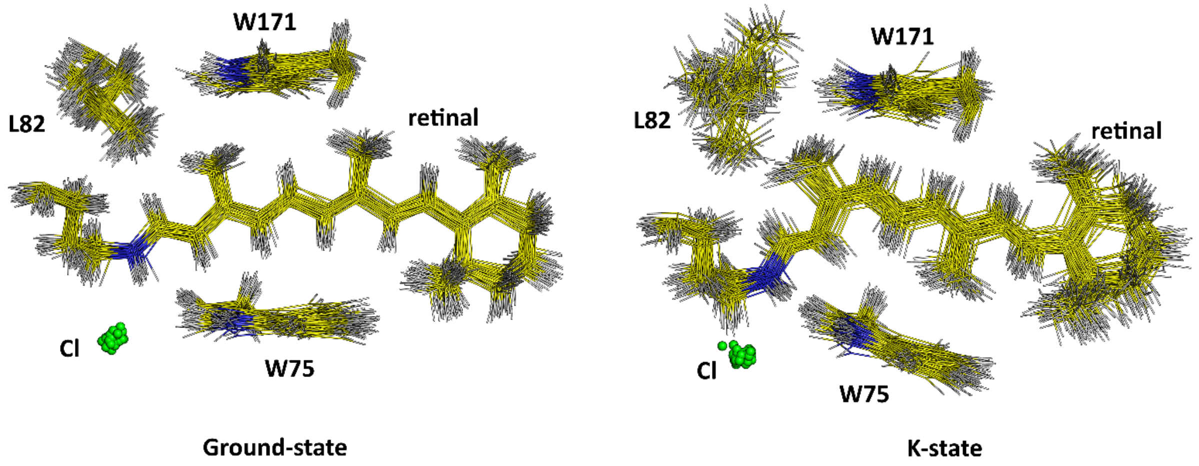

Supplementary Fig. 7 | Molecular dynamics simulation of chloride-bound *Sy*HR in the ground (left) and K (right) states. 50 snapshots taken each 4 ns are shown. The retinal, chloride ion, water molecules and neighboring side chains maintain their positions throughout the simulations.

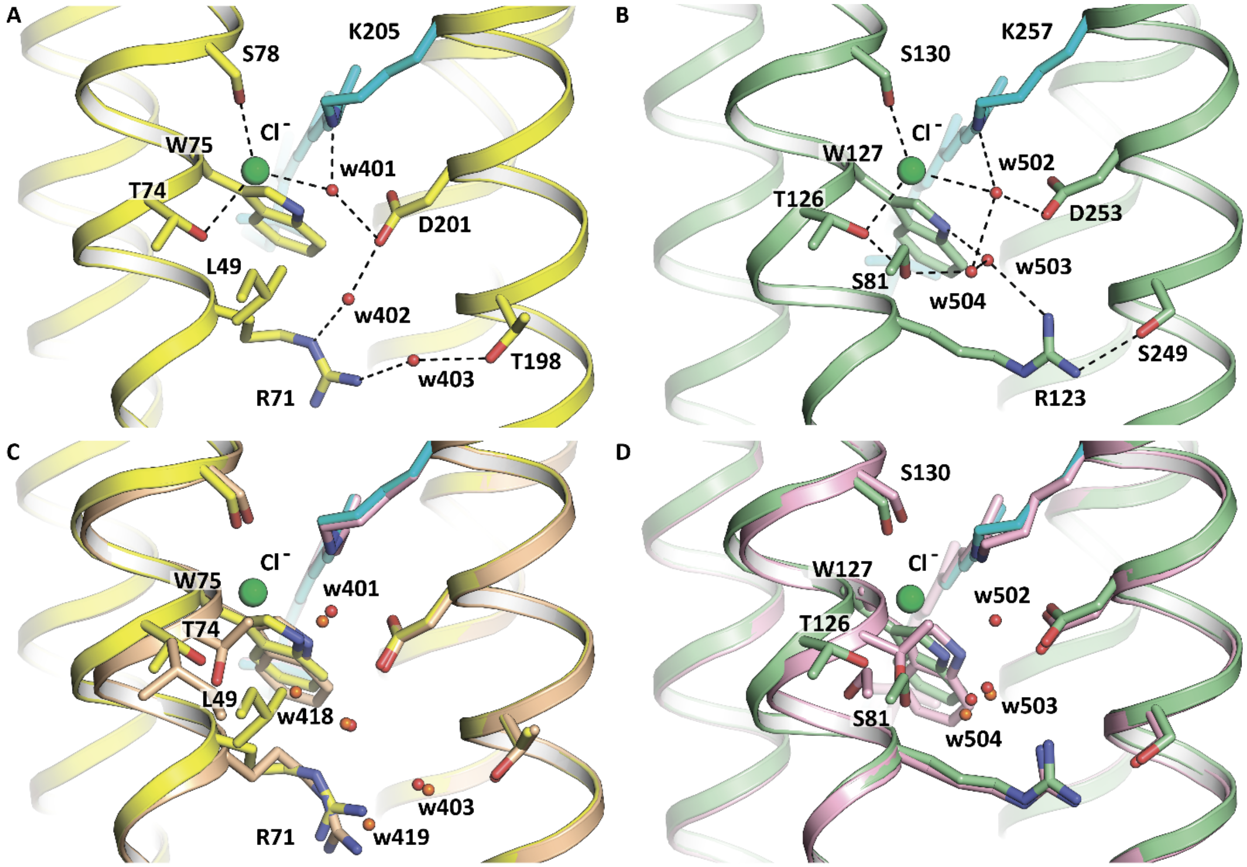

Supplementary Fig. 8 | Structure of the RSB region in *Sy*HR and *Np*HR. (A) Structure of the Cl^--bound form of *Sy*HR. (B) Structure of Cl^--bound form of *Np*HR (PDB ID: 3A7K). (C) Comparison between the ground state (yellow protein and red waters) and the O-state (wheat protein and orange waters) of *Sy*HR. The cofactor retinal and K205 are colored cyan in the ground state structure of *Sy*HR. (D) Comparison between the ground state (green protein and red waters) and the O-like anion free form (pink protein and orange waters) of *Np*HR (PDB ID: 3QBG). The cofactor retinal and K205 are colored cyan in the ground state structure of *Sy*HR.

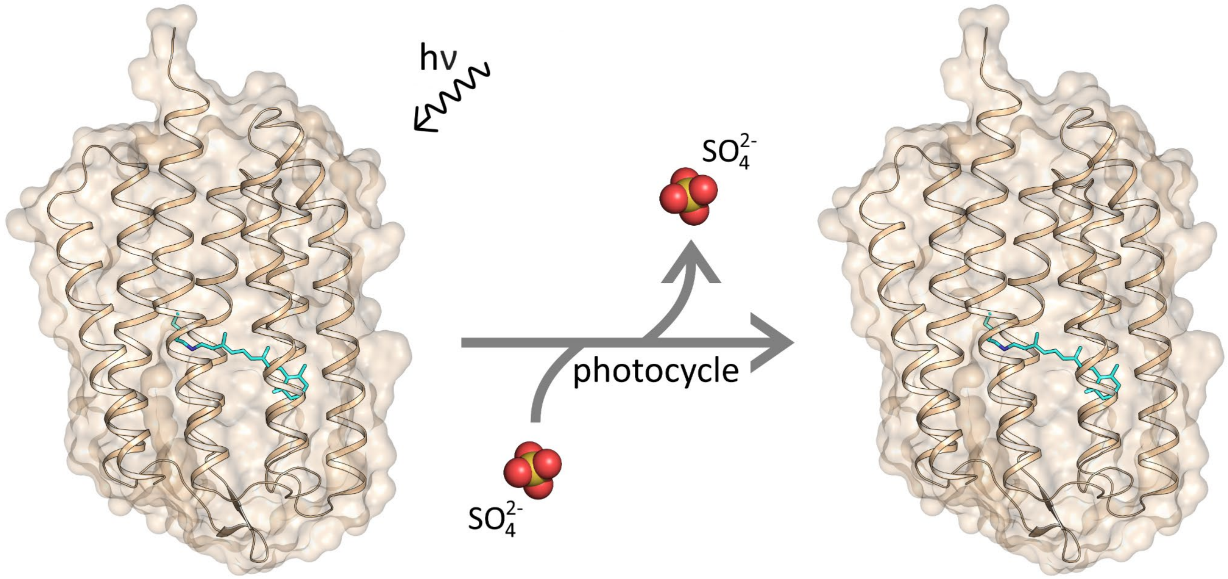

Supplementary Fig. 9 | Schematic representation of sulfate pumping process by *Sy*HR. Sulfate ion enters the inner space of the protein after the beginning of photocycle and come out before the end of photocycle.

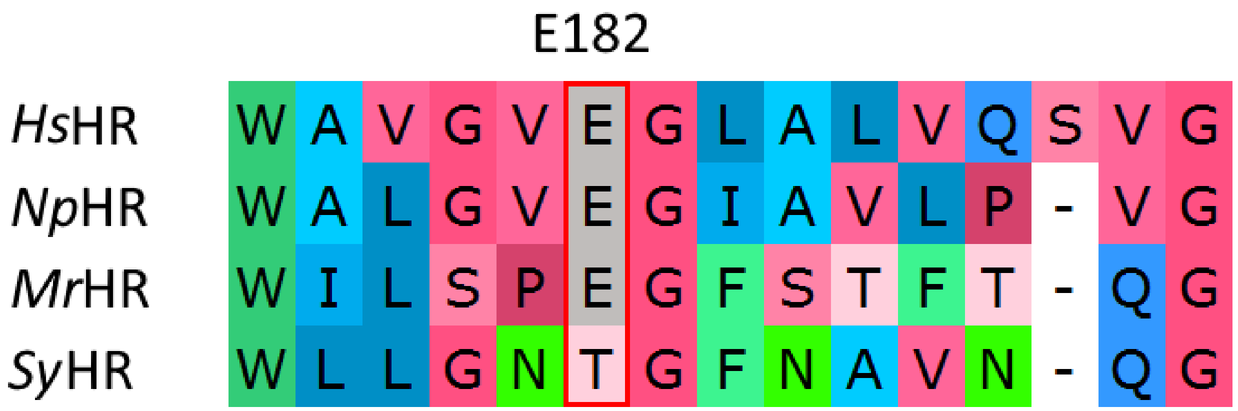

Supplementary Fig. 10 | Sequence alignment of chloride pumping rhodopsins in the E182 region.

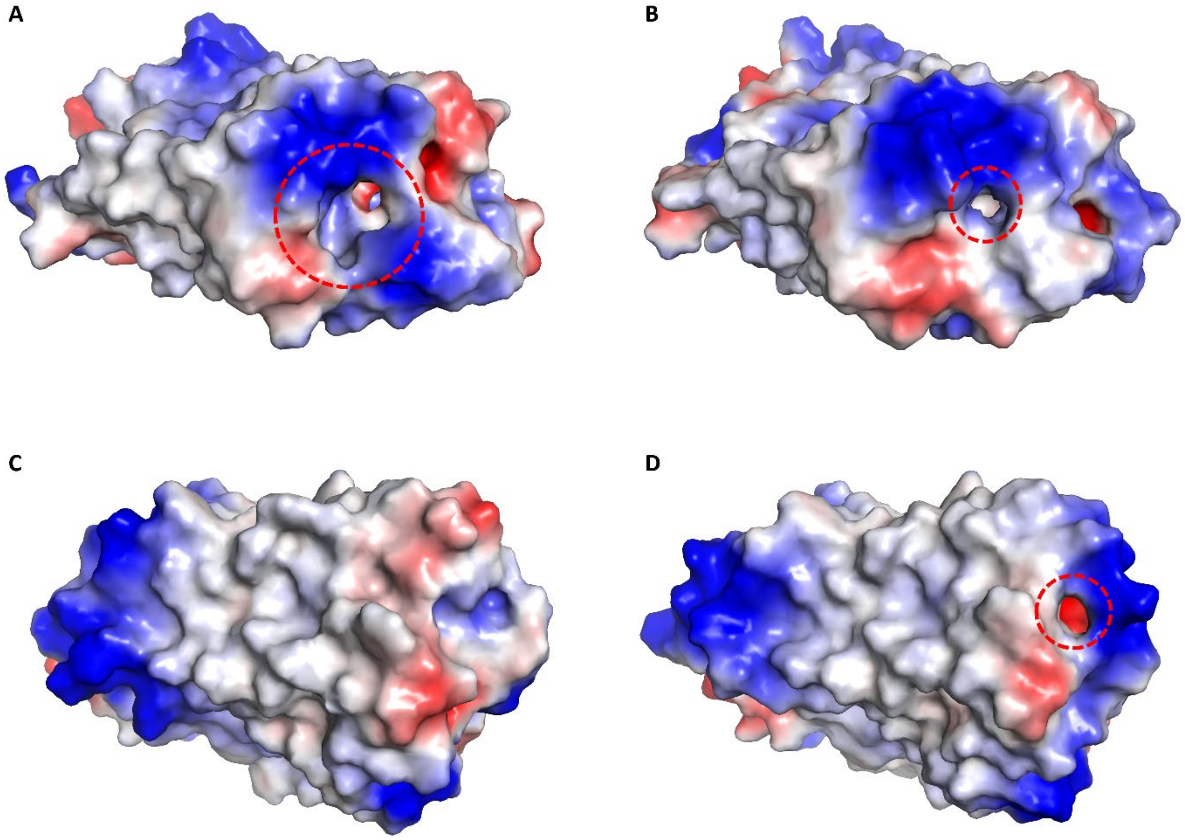

Supplementary Fig. 11 | Putative anion entrance at the extracellular side of cyanobacterial HRs. (A) The main putative entrance to the extracellular cavity in *Sy*HR. (B) The main putative entrance to the extracellular cavity in *Mr*HR (PDB ID: 6K6I). (C) The second way for sulfate to enter the protein, obstructed in *Sy*HR. (D) The second way for sulfate to enter the protein, opened in *Mr*HR-N63A/P118A (PDB ID: 6K6K).

Supplementary Tables

Supplementary Table 1. Data collection and refinement statistics.

**Data collection**	Ground Cl^-	Ground SO_4^{2-}	K state	O state
**PDB ID**	7ZOU	7ZOV	7ZOW	7ZOY
Space group	P 3 2 1	P 3 2 1	P 3 2 1	P 3 2 1
**Cell dimensions**				
a, b, c (Å)	61.98, 61.98, 109.83	62.30, 62.30, 110.14	61.99, 61.99, 109.82	62.41, 62.41, 110.08
α, β, γ (°)	90, 90, 120	90, 90, 120	90, 90, 120	90, 90, 120
Resolution (Å)	53.68 - 1.58 (1.61-1.58)	48.45 - 1.91 (1.94 - 1.91)	48.23 - 1.70 (1.73 - 1.70)	48.52 - 1.60 (1.64-1.60)
Anisotropic resolution limits (Å)	1.54, 1.54, 1.62	1.87, 1.87, 1.89	1.67, 1.67, 1.70	1.56, 1.56, 1.71
No. of observations	352260 (7541)	211230 (8597)	294113 (14721)	349218 (17878)
No. of unique reflections	33368 (1667)	19823 (992)	27378 (1379)	31399 (1572)
Rpim	0.059 (0.993)	0.081 (0.816)	0.101 (0.975)	0.065 (0.820)
I/σI	8.0 (0.7)	7.5 (0.9)	5.5 (0.9)	7.0 (1.1)
CC1/2	99.8 (22.5)	99.7 (39.9)	99.6 (30.6)	99.9 (41.0)
Completeness spherical (%)	96.9 (72.8)	99.6 (92.3)	99.2 (91.7)	93.8 (62.3)
Completeness anisotropic (%)	98.2 (90.3)	99.6 (92.3)	99.2 (91.7)	97.5 (93.8)
Multiplicity	10.6 (4.5)	10.7 (8.7)	10.7 (10.7)	11.1 (11.4)
Wilson B-factor	17.56	22.36	18.76	22.21
**Refinement statistics**				
Resolution (Å)	19.95 - 1.58	19.27 - 1.91	19.95 - 1.70	19.28 - 1.60
No. unique reflections	33331	19758	27263	31363
Rwork/Rfree	0.1735/0.2023	0.1946/0.2280	0.1849/0.2217	0.1950/0.2316
No. of atoms				
Protein	1901	1923	1932	1889
Retinal	20	20	40	20
Cl	1	1	1	0
SO_4	0	5	0	0
Water	126	76	126	122
Lipids	238	165	238	233
No. of TLS groups	4	0	0	4
B-factors (Å^2)				
Protein	19.89	25.25	19.86	20.03
Retinal	13.89	20.19	14.87	15.35
Cl	14.51	19.32	15.88	-
SO_4	-	62.59	-	-
Water	34.00	34.03	33.44	32.43

Lipids	41.00	43.04	41.94	39.93
R.m.s.d.				
Bong lengths (Å)	0.005	0.011	0.006	0.006
Bond angles (°)	0.84	1.52	0.82	0.87
Ramachandran statistics				
Favoured (%)	99.09	99.09	99.09	98.63
Allowed (%)	0.91	0.91	0.91	1.37

Supplementary Table 2. Root mean square fluctuations of side chain heavy atom positions in simulations of chloride-bound *Sy*HR in the ground and K states, Å.

	Ground	K
K205 and retinal	0.285 ± 0.004	0.439 ± 0.017
L82	0.306 ± 0.014	1.204 ± 0.050
W75	0.307 ± 0.011	0.294 ± 0.009
W171	0.378 ± 0.022	0.383 ± 0.031

Supplementary Data.

Supplementary Data 1. Atomistic model of the starting structure used in molecular dynamics simulations of the sulfate-bound *Sy*HR trimer.

Supplementary Data 2. Average densities of sulfate sulfur atoms obtained in molecular dynamics simulations.
