# Supplementary material for: Structural insights into light-driven anion pumping in cyanobacteria
Source: Nat Commun. 2022 Oct 29;13:6460. doi: 10.1038/s41467-022-34019-9 (PMC9617919; doi:10.1038/s41467-022-34019-9)
Supplement: Supplementary file 3 — Description of Additional Supplementary Files [file 41467_2022_34019_MOESM3_ESM.pdf]

File Name: Supplementary Data 1

Description: Atomistic model of the starting structure used in molecular dynamics simulations of the sulfate-bound *Sy*HR trimer.

File Name: Supplementary Data 2

Description: Average densities of sulfate sulfur atoms obtained in molecular dynamics simulations.
