# Supplementary material for: Structural insights into light-driven anion pumping in cyanobacteria
Source: Nat Commun. 2022 Oct 29;13:6460. doi: 10.1038/s41467-022-34019-9 (PMC9617919; doi:10.1038/s41467-022-34019-9)
Supplement: Supplementary file 6 — Reporting Summary [file 41467_2022_34019_MOESM6_ESM.pdf]

nature portfolio

Corresponding author(s): Valentin Gordeliy

Last updated by author(s): Sep 22, 2022

Reporting Summary

Nature Portfolio wishes to improve the reproducibility of the work that we publish. This form provides structure for consistency and transparency in reporting. For further information on Nature Portfolio policies, see our Editorial Policies and the Editorial Policy Checklist.

Statistics

For all statistical analyses, confirm that the following items are present in the figure legend, table legend, main text, or Methods section.

n/a	Confirmed	
☒	☐	The exact sample size (*n*) for each experimental group/condition, given as a discrete number and unit of measurement
☐	☒	A statement on whether measurements were taken from distinct samples or whether the same sample was measured repeatedly
☒	☐	The statistical test(s) used AND whether they are one- or two-sided<br>*Only common tests should be described solely by name; describe more complex techniques in the Methods section.*
☒	☐	A description of all covariates tested
☒	☐	A description of any assumptions or corrections, such as tests of normality and adjustment for multiple comparisons
☐	☒	A full description of the statistical parameters including central tendency (e.g. means) or other basic estimates (e.g. regression coefficient) AND variation (e.g. standard deviation) or associated estimates of uncertainty (e.g. confidence intervals)
☒	☐	For null hypothesis testing, the test statistic (e.g. *F*, *t*, *r*) with confidence intervals, effect sizes, degrees of freedom and *P* value noted<br>*Give P values as exact values whenever suitable.*
☒	☐	For Bayesian analysis, information on the choice of priors and Markov chain Monte Carlo settings
☒	☐	For hierarchical and complex designs, identification of the appropriate level for tests and full reporting of outcomes
☒	☐	Estimates of effect sizes (e.g. Cohen's *d*, Pearson's *r*), indicating how they were calculated

Our web collection on statistics for biologists contains articles on many of the points above.

Software and code

Policy information about availability of computer code

Data collection	MxCube was used for diffraction data collection
Data analysis	XDS package (BUILT=20210205) for crystallography processing; CCP4i program suite (v7.1.018) for structure solving; PHENIX program suite (v1.19.2-4158) for structure refinement; PyMoL (v1.8.4.0) for structure visualization; OriginPro (v9.85) for spectra visualization; Coot (v0.9.6) for structure refinement; iTol v6 online server for phylogenetic tree generation; Wolfram Mathematica (v11) for bioinformatic analysis; Jupyter Notebook (v5.2.2) for bioinformatic analysis; Python (v3.6.9) for bioinformatic analysis; CNS (v1.3) for difference electron density maps building; refmac5 (v5.8.0267) for structure refinement; GROMACS2022 for the MD simulations.

For manuscripts utilizing custom algorithms or software that are central to the research but not yet described in published literature, software must be made available to editors and reviewers. We strongly encourage code deposition in a community repository (e.g. GitHub). See the Nature Portfolio guidelines for submitting code & software for further information.

Data

Policy information about availability of data

All manuscripts must include a data availability statement. This statement should provide the following information, where applicable:

- Accession codes, unique identifiers, or web links for publicly available datasets
- A description of any restrictions on data availability
- For clinical datasets or third party data, please ensure that the statement adheres to our policy

Atomic models built using X-ray crystallography data have been deposited in the RCSB Protein Data Bank with PDB codes 7Z0U (for the ground state of Cl-bound SyHR), 7Z0V (for the ground state of sulfate-bound SyHR, 7Z0W (for the K state of SyHR) and 7Z0Y (for the O state of SyHR). The Uniprot gene database (uniprot.org) was used for bioinformatic analysis of the light-driven ion transporters group of microbial rhodopsins.

Human research participants

Policy information about studies involving human research participants and Sex and Gender in Research.

Reporting on sex and gender	The work did not include human research
Population characteristics	*Describe the covariate-relevant population characteristics of the human research participants (e.g. age, genotypic information, past and current diagnosis and treatment categories). If you filled out the behavioural & social sciences study design questions and have nothing to add here, write "See above."*
Recruitment	*Describe how participants were recruited. Outline any potential self-selection bias or other biases that may be present and how these are likely to impact results.*
Ethics oversight	*Identify the organization(s) that approved the study protocol.*

Note that full information on the approval of the study protocol must also be provided in the manuscript.

Field-specific reporting

Please select the one below that is the best fit for your research. If you are not sure, read the appropriate sections before making your selection.

☒ Life sciences ☐ Behavioural & social sciences ☐ Ecological, evolutionary & environmental sciences

For a reference copy of the document with all sections, see nature.com/documents/nr-reporting-summary-flat.pdf

Life sciences study design

All studies must disclose on these points even when the disclosure is negative.

Sample size	We did not use statistical methods to predetermine sample size. The sample size for X-ray studies (number of crystals used for cryotrapping of the intermediates and structure determinations) was 5 or greater.
Data exclusions	No data were excluded.
Replication	The cryotrapping procedures were repeated no less than 5 times for each of the intermediate states. At least two datasets were collected for each state of the SyHR presented in this study and only the highest-resolution/highest quality data is presented in the manuscript.
Randomization	The samples were not allocated into different groups, therefore the randomization was not required.
Blinding	Animal experiments were not performed in the study, so the Investigators were not blinded to the experiments. No other experiments in this study involve blinding.

Reporting for specific materials, systems and methods

We require information from authors about some types of materials, experimental systems and methods used in many studies. Here, indicate whether each material, system or method listed is relevant to your study. If you are not sure if a list item applies to your research, read the appropriate section before selecting a response.

Materials & experimental systems

n/a	Involved in the study
☒	☐ Antibodies
☒	☐ Eukaryotic cell lines
☒	☐ Palaeontology and archaeology
☒	☐ Animals and other organisms
☒	☐ Clinical data
☒	☐ Dual use research of concern

Methods

n/a	Involved in the study
☒	☐ ChIP-seq
☒	☐ Flow cytometry
☒	☐ MRI-based neuroimaging
